# Impact on hospital mortality and morbidity of right ventricular involvement among patients with acute left ventricular infarction

**DOI:** 10.1590/S1516-31802006000400003

**Published:** 2006-05-04

**Authors:** Afonso Celso Pereira, Roberto Alexandre Franken, Sandra Regina Schwarzwälder Sprovieri, Valdir Golin

**Keywords:** Myocardial infarction, Heart ventricles, Right ventricular dysfunction, Hospital mortality, Morbidity, Infarto do miocárdio, Ventrículos cardíacos, Disfunção ventricular direita, Mortalidade hospitalar, Morbidade

## Abstract

**CONTEXT AND OBJECTIVE:**

There is uncertainty regarding the risk of major complications in patients with left ventricular (LV) infarction complicated by right ventricular (RV) involvement. The aim of this study was to evaluate the impact on hospital mortality and morbidity of right ventricular involvement among patients with acute left ventricular myocardial infarction.

**DESIGN AND SETTING:**

Prospective cohort study, at Emergency Care Unit of Hospital Central da Irmandade da Santa Casa de Misericórdia de São Paulo.

**METHODS:**

183 patients with acute myocardial infarction participated in this study: 145 with LV infarction alone and 38 with both LV and RV infarction. The presence of complications and hospital death were compared between groups.

**RESULTS:**

21% of the patients studied had LV + RV infarction. In this group, involvement of the dorsal and/or inferior wall was predominant on electrocardiogram (p < 0.0001). The frequencies of Killip class IV upon admission and 24 hours later were greater in the LV + RV group, along with electrical and hemodynamic complications, among others, and death. The probability of complications among the LV + RV patients was 9.7 times greater (odds ratio, OR = 9.7468; 95% confidence interval, CI: 2.8673 to 33.1325; p < 0.0001) and probability of death was 5.1 times greater (OR = 5.13; 95% CI: 2.2795 to 11.5510; p = 0.0001), in relation to patients with LV infarction alone.

**CONCLUSIONS:**

Patients with LV infarction with RV involvement present increased risk of early morbidity and mortality.

## RESUMO

**CONTEXTO E OBJETIVO:**

O risco das principais complicações em pacientes com infarto do ventrículo esquerdo (VE) associado ao comprometimento do ventrículo direito (VD) ainda é incerto. O objetivo deste estudo foi avaliar o impacto do envolvimento do ventrículo direito na morbidade e na mortalidade hospitalar em pacientes com infarto agudo do ventrículo esquerdo.

**TIPO DE ESTUDO E LOCAL:**

Estudo prospectivo de coorte, realizado no Serviço de Emergência do Hospital Central da Irmandade da Santa Casa de Misericórdia de São Paulo.

**MÉTODOS:**

Participaram do estudo 183 pacientes com infarto agudo do miocárdio: 145 com infarto do VE e 38 com infarto do VE associado ao VD (VE + VD). Foram comparados entre os grupos a presença das complicações e óbitos no período hospitalar.

**RESULTADOS:**

21% dos pacientes estudados apresentavam infarto VE + VD. Predominou o comprometimento da parede dorsal e/ou inferior (ECG) no grupo com VE + VD (p < 0.0001). A freqüência da classe IV de Killip no momento da admissão e 24 h após foi maior no grupo VE + VD, assim como as complicações elétricas, hemodinâmicas, entre outras, e óbitos. A probabilidade de complicações em pacientes com infarto VE + VD foi 9.7 vezes maior (*odds ratio,* OR = 9.7468; 95% intervalo de confiança, IC 95% 2.8673 a 33.1325; p < 0.0001) e de óbito 5.1 vezes superior (OR = 5.1313; 95% IC 2.2795 a 11.5510; p = 0.0001) em relação aos pacientes com infarto isolado do VE.

**CONCLUSÕES:**

Pacientes com infarto VE com envolvimento do VD apresentam risco aumentado de morbidade e mortalidade precoces.

## INTRODUCTION

Approximately 50% of inferior wall infarction cases are associated with right ventricular (RV) infarction.^1,2^ The incidence of left ventricular (LV) infarction in association with RV infarction ranges from 14% to 84%.^3-5^ Infarction of the right ventricle alone, on the other hand, is reported in less than 3% of myocardium infarction (MI) cases.^6,7^ From an epidemiological point of view, these differences make it difficult to accurately evaluate the impact of RV infarction in populations studied.

It is the right coronary artery that is most frequently involved, obstructed in its proximal third, thus resulting in dysfunction of the free walls of the right ventricle and the walls of the inferior left ventricle.^8^ Although the performance of the right ventricle spontaneously improves even in the absence of coronary reperfusion, its recovery may be slow and may be associated with high rates of atrioventricular conduction, hemodynamic instability and hospital mortality.^9–11^ Reperfusion optimizes the recuperation of the right ventricle and improves patients’ clinical evolution.^8^

The gold standard for the diagnosing of RV infarction is hemodynamic evaluation or an autopsy. It is known that this diagnosis may be made by clinical evaluation,^12,13^ electrocardiogram (EKG), hemodynamic evaluation, studies using radioisotopes such as technetium 99m (^99m^Tc) pyrophosphate, and right nuclear ventriculography.

EKG using the right V_3_ and V_4_ (V_3_R and V_4_R) derivations is a simple method presenting good sensitivity and specificity.^14–17^ Identification of right ventricular abnormality, shown by elevation of the ST segment in the right derivations, especially V_4_R, is an important predictor of hospital complications and mortality.^17^

It is now known that thrombolytic therapy in patients presenting infarction in the inferior wall is beneficial for reducing infarct magnitude and mortality.^8^ Patients with inferior myocardial infarction who have RV myocardial involvement appear to have a worse prognosis than those who do not have RV involvement. These patients appear to be at increased risk of severe complications, such as left ventricular failure, cardiogenic shock and serious ventricular arrhythmia, and even death.^18^

The annual mortality rate for patients with acute myocardial infarction affecting the right ventricle is 4.2%.^17^ Data in the literature demonstrate that the only independent predictive factors for hospital mortality are advanced age and RV infarction, with a significant mortality rate among patients over the age of 80 years. However, this is not so for patients who are under the age of 40 years.^18^

## OBJECTIVE

The aim of this study was to evaluate the prognostic impact of RV myocardial involvement in patients with acute LV myocardial infarction, comparing the frequency of hospital morbidity and mortality among patients with LV + RV infarction and those with LV infarction alone.

## METHODS

From January 1998 to September 2000, we prospectively studied 192 consecutive patients who were admitted to the emergency care unit of Hospital Central da Irmandade da Santa Casa de Misericórdia de São Paulo with a diagnosis of acute MI. All patients were 18 years of age or older and had their definite diagnosis established when at least two of the following criteria were present at admission: (1) within 48 hours of an episode of ischemic chest pain at rest lasting for ≥ 30 minutes; (2) for the left ventricle, presence of an ST-segment elevation ≥ 0.1 mV in two or more of the classic derivations, or 0.2 mV elevation in two or more contiguous precordial derivations, or left branch block on EKG, and/or appearance of an R wave in V1 or V2 > 0.04 second with an R/S voltage ratio > 1, and for the right ventricle, presence of an ST-segment elevation ≥ 0.1 mV in the V_3_R and V_4_R derivations; (3) elevation of the serum creatine kinase (CK) level to at least twice the normal upper limit (150 U/l in our institution), with an MB fraction (CK-MB) > 10% of the total CK level.

All of the patients suspected of having acute MI were routinely submitted to 16-lead EKG (12 standard leads plus two right pre-cordial leads, V_3_R and V_4_R, and two posterior leads, V_7_ and V_8_). The EKG criteria were analyzed by two different observers. Patients suspected of having MI who presented factors that reduced the sensitivity of the EKG criteria, such as pericarditis, pulmonary embolism, anterior-superior divisional block and prior infarcts, were excluded. Six patients for whom the presence of LV involvement could not be determined using the EKG criteria that had been defined, and two patients with chest pain that was not of confirmed cardiac origin, were excluded.

The local Ethics Committee gave prior approval for this study, and informed consent was obtained from the patients that participated in the study. One patient did not participate because this person disagreed with the protocol. The final study group thus consisted of 183 patients.

The patients were grouped into two categories for comparisons:

(1) LV: 145 patients of both genders, with left ventricular acute myocardial infarction alone.

(2) LV + RV: 38 patients of both genders, with both left and right ventricular acute myocardial infarction.

The patients were also grouped into two age categories: 65 years old or less and over 65 years. The time elapsed between the beginning of the symptoms and hospital admission was analyzed in terms of the following periods: up to 60 minutes; more than 60 minutes and up to 4 hours; more than 4 hours and up to 12 hours; and more than 12 hours. In order to evaluate the severity of the acute MI, the classification proposed by Killip & Kimball^19^ was adopted for each case at the time of admission and 24 hours after hospitalization. The walls of the left ventricle involved were classified as anterior, inferior and/or dorsal, and non-Q wave infarction.

The study patients did not undergo emergency hemodynamic tests. Thrombolytic treatment using intravenous streptokinase was administered to all patients admitted up to 12 hours from when the thoracic pain began who were ≤ 75 years old and presented with absence of other contraindications.^20^ In cases in which coronariography was done, this procedure was performed around the seventh day after hospital admission, in accordance with the routine in our service. The coronary arteries considered related to the event were the left anterior descending artery (LAD), circumflex artery (CX) or the right coronary artery (RCA), and there was also an “unidentifiable” category.

The groups were evaluated with regard to the complications from the acute myocardial infarction:

- Electrical: evolution with second-degree atrioventricular block, total atrioventricular block, sustained ventricular tachycardia, ventricular fibrillation, atrial fibrillation, atrial flutter or paroxysmal supraventricular tachycardia.
- Hemodynamic: evolution with arterial hypotension (systolic blood pressure < 90 mmHg) in the absence of vasodilator use, which was corrected with infusion of 2,000 ml of physiological solution during the first 24 hours; or left cardiac insufficiency, acute pulmonary edema or cardiogenic shock.
- Other complications: those related to acute MI, such as systemic embolism, stroke, hemorrhagic cerebrovascular accident, pulmonary embolism, deep vein thrombosis, re-infarction and infections.

### Statistical analysis

Means ± standard deviation (SD) were calculated for continuous variables. Differences between groups were examined for statistical significance using the chi-squared test (χ^2^), with Yates correction when applicable, for comparisons between the proportions of categorical variables. Discrete categorical variables were compared using Fisher's exact test. A 2 x 2 table was formed by considering each of the complications versus all other complications, and this was done in the same manner to analyze the coronary artery responsible for the event. The risk of complications and death was calculated using odds ratios (OR) with a confidence interval of 95% (95% CI). Values were considered to be statistically significant when p < 0.05.

## RESULTS

Of the 183 patients with acute MI, 21% had LV + RV and 79% had LV infarction alone. There was a predominance of the male gender (72.4% versus 57.9% for the LV and LV + RV groups, respectively), although with no significant difference between the groups. The mean age of the patients with LV infarction was 60.1 ± 2.4 years and, for those with LV + RV, it was 60.2 ± 12.4 years. Due to the predominance of patients aged less than or equal to 65 years in both groups, no difference was found between the groups when the age groups were compared (χ^2^ = 0.415; p = 0.652) (Table 1).

**Table 1.** Baseline characteristics and hospital evolution of 183 consecutive patients with acute myocardial infarction: 145 with left ventricle infarction alone and 38 with both left and right ventricular infarction

Characteristics	LV (n = 145)	LV + RV (n = 38)	Total (n = 183)	p value
Sex (male/female)	105/40	22/16	127/56	NS
Age (years)				
≤ 65	95 (65%)	27 (71%)	122 (67%)	NS
> 65	50 (35%)	11 (29%)	61 (33%)	
Time elapsed between symptom onset and hospital admission				
Up to 1 h	34 (23%)	12 (32%)	46 (25%)	NS
> 1 h – 4 h	49 (34%)	15 (39%)	64 (35%)	
> 4 h – 12 h	38 (26%)	9 (24%)	47 (26%)	
> 12 h	24 (17%)	2 (5%)	26 (14%)	
Wall involved (on EKG)				
Anterior	90 (62%)*	7 (18%)	97 (53%)	< 0.0001
Inferior and/or dorsal	43 (30%)	31 (82%)*	74 (40%)	
Non-Q wave infarction	12 (8%)	0	12 (7%)	
Killip class upon hospital admission				
I	117 (81%)*	20 (53%)	137 (75%)	< 0.0001
II	19 (13%)	8 (21%)	27 (15%)	
III	5 (3%)	0	5 (3%)	
IV	4 (3%)	10 (26%)*	14 (7%)	
Killip class 24 hours after admission				
I	101 (70%)*	15 (43%)	116 (64%)	< 0.0001
II	26 (18%)	6 (17%)	32 (18%)	
III	6 (4%)	0	6 (3%)	
IV	12 (8%)	14 (40%)*	26 (15%)	

*The chi-squared and Fisher exact tests were used when applicable. LV = left ventricular infarction alone. LV + RV = both left and right ventricle involvement. Three patients with LV + RV infarction died within the first 24 hours following hospital admission. NS = not significant, i.e. p > 0.05. EKG = electrocardiogram.*

* *p < 0.05.*

The time elapsed between symptom onset and hospital admission was similar for the two groups, with predominance of the interval between one and four hours (χ^2^ = 3.845; p = 0.279) (Table 1). There was no noteworthy difference concerning the distribution of patients by age, gender and time elapsed between symptom onset and hospital admission in either group.

The EKG results showed predominance of anterior wall infarction in the group with LV alone (χ^2^ = 23.028; p < 0.0001), while in the LV + RV group there was greater damage to the inferior and/or dorsal wall (χ^2^ = 33.704; p < 0.0001) (Table 1).

Killip class I was more frequently found in the group of patients with LV infarction than in the group with LV + RV infarction, both at the time of admission and after 24 hours of acute MI evolution (χ^2^ = 12.596, p = 0.008; and χ^2^ = 8.836, p = 0.005; upon admission and after 24 hours, respectively), while Killip class IV was more frequently found in the LV + RV group than in the LV group, at both times (χ^2^ = 23.65, p < 0.0001; and χ^2^ = 22.961, p < 0.0001; upon admission and after 24 hours, respectively). Sixteen patients with LV + RV infarction progressed to death (Table 2), three of them within 24 hours of admission to the hospital.

**Table 2.** Mortality and other complications during the hospitalization period among 183 patients with acute myocardial infarction

Characteristics	LV (n = 145)	LV + RV (n = 38)	Total (n = 183)	P value
Complications	with/without	with/without	with/without	
Hemodynamic	55/90	27/11*	82/101	0.0005
Electrical	43/102	22/16*	65/118	0.0023
Others	25/120	21/17*	46/137	< 0.0001
Evolution				
Released	127 (88%)*	22 (58%)	149 (81%)	0.0001
Death	18 (12%)	16 (42%)*	34 (19%)	

*The chi-squared and Fisher exact tests were used when applicable. Complications were present in 79/145 patients with LV infarction and 35/38 patients with LV + RV infarction. Some of the patients had more than one complication. LV = left ventricular infarction alone. LV + RV = both left and right ventricle involvement.*

* *p < 0.05.*

The indication for thrombolytic therapy was similar for the two groups, and was instituted for 65% (94/145) of the patients with LV alone and 76% (29/38) of those with LV + RV infarction (χ^2^ = 1.803; p = 0.251).

Of the 107 patients that underwent coronariography (89 with LV and 18 with LV + RV), it was observed that the left anterior descending artery (LAD) was the coronary artery most frequently affected in LV infarction (53% versus 11%; χ^2^ = 11.32, p = 0.0016), while abnormalities of the right coronary artery (RCA) were more predominant in the group with LV + RV (83% versus 22%; χ^2^ = 12.838, p = 0.0008).

The length of hospitalization was similar for the two groups: 9.6 ± 7.6 days in the group with LV alone, and 9.8 ± 9.0 days in the group with LV + RV (95% CI: 0.3117 to 0.2277; p = 0.8647).

During the hospitalization period, 62% of the patients with MI presented complications, and this frequency was significantly greater among patients with LV + RV infarction (92% versus 54%; χ^2^ = 18.144, p < 0.0001) (Table 2). It was observed that the incidence of complications among the patients with LV + RV infarction was 9.7 times greater than for the patients with LV alone (odds ratio, OR = 9.7468; 95% CI: 2.8673 to 33.1325; p < 0.0001).

Analysis of the electrical complications showed that these were significantly more frequent in the group of patients with LV + RV than in the group with LV infarction alone (58% versus 30%, respectively; χ^2^ = 13.356, p = 0.0005). This complication was absent in the majority of patients with LV infarction classified as Killip class I upon hospital admission and after 24 hours. Patients who were not administered thrombolytics evolved with a greater number of electrical complications than those that received the medication (for LV: 22/43 patients, χ^2^ =5.006, p = 0.041; and for RV: 14/22 patients, χ^2^ = 4.647, p = 0.031). The presence of arrhythmia did not correlate with progression to death in either group.

Hemodynamic complications were also significantly more frequent in the patients with LV + RV infarction (71% versus 38%; χ^2^ = 10.484, p = 0.0023). The presence of hemodynamic complications did not demonstrate any association with age in the LV + RV group, yet was significantly more frequent in patients over the age of 65 years in the group with LV alone (16/27 patients, χ^2^ = 6.307, p = 0.043). Neither group demonstrated correlation between hemodynamic complications and the parameters of gender, time elapsed from symptom onset to hospital admission, Killip class, administration of thrombolytics, or progression to death.

Complications other than electrical or hemodynamic ones were also more frequent in the group with LV + RV infarction (55% versus 17%; χ^2^ = 23.131, p < 0.0001), and these other complications did not exhibit any important correlation with the patients’ ages, gender, time elapsed from symptom onset to hospital admission, Killip class, the introduction of thrombolytics, or even progression to death.

There was no association between the presence of electrical or hemodynamic complications, among others, and the different locations of the infarct of the LV wall, on the EKG, or with regard to the coronary arteries responsible for the event.

The incidence of death was significantly higher in the group with LV + RV infarction (42% versus 12%; χ^2^ = 17.547, p = 0.0001) (Table 2). The risk of death was 5.1 times greater among patients with LV + RV than among those with LV infarction alone (OR = 5.1313; 95% CI: 2.2795 to 11.5510; p = 0.0001).

Mortality was significantly more frequent in patients over the age of 65 in both groups. It became evident that Killip class IV at the time of admission and also after 24 hours of acute MI evolution was intimately related to progression to death in both groups. There was no observed association between non-introduction of thrombolytics and progression to death in the group with LV infarction alone, while the patients with both LV + RV infarction who did not receive the medication progressed to death more frequently. No significant associations were found for the location on the LV wall (as seen on EKG) or the affected coronary artery, in relation to progression to death in either group.

## DISCUSSION

The present study undertaken in a tertiary level hospital in the southeastern region of Brazil had the goal of corroborating the ever-growing evidence that infarction of the left ventricle in association with right ventricular damage significantly interferes in the evolution of patients with acute MI. The publication of several scientific studies regarding this subject has alerted physicians to the need for early diagnosis and immediate recognition of the possible clinical complications. These studies have also provided information about more effective treatment, with the intention of providing better prognosis for these patients.^8,21-24^

The present study has demonstrated that 21% of the patients with LV myocardial infarction presented this in association with RV infarction. This is in accordance with the data in the literature, with reported percentages ranging from 14% to 84%.^3-5,25,26^ Among such cases, 82% were associated with damage of the inferior-dorsal wall of the left ventricle. This was a greater proportion than in the literature, with reported frequencies for this association of around 50%.^1,2^ Damage to the right coronary artery was present in 83% of the cases we studied. RV infarction alone was not detected in our study.

In both groups, the male gender predominated in the age range of up to 65 years, i.e. the male patients in our study were younger than those in the literature.^5,26^ Although the variable “time elapsed between symptom onset and hospital admission” cannot be considered to be a direct predictive factor regarding patient evolution, it has already been observed in a study conducted by Franken et al.^27^, in our institution, that 30% of myocardial infarction cases, regardless of the affected ventricle, arrive at the hospital six hours after symptom onset.

Despite the clinical severity (Killip class IV) that affected the great majority of the group with LV + RV infarction at the time of hospital admission, 76% of these patients received thrombolytic treatment with the aim of improving their evolution. Even so, the complications that affected 92% of these patients were unavoidable.

All of these complications, regardless of age, gender and time elapsed until hospital arrival, were present in the group that presented RV involvement, while hemodynamic complications were most frequently found in the patients with LV infarction alone who were 65 years of age or older.

The use of thrombolytics reduced the occurrence of electrical complications in both groups, yet did not reduce the hemodynamic and other complications of the group with LV + RV infarction. César et al.,^28^ in a study on the hemodynamic behavior of the right ventricle in relation to infarction of the inferior wall, concluded that evolution to cardiogenic shock depended mainly on right ventricle insufficiency, independent of whether left ventricular function was preserved.

The present study demonstrated that 42% of the patients with LV + RV infarction progressed to death during the hospitalization period, whereas among those who presented LV alone, 12% died. It was also demonstrated that some baseline characteristics, such as patient age over 65 years, presence of Killip class IV and no use of thrombolytics, were associated with mortality among patients with RV involvement in acute MI infarction of the LV.

Considering similar data, Bowers et al.^8^ suggested that successful reperfusion by means of primary angioplasty could achieve dramatic improvements in RV performance and improved clinical outcomes among patients with RV myocardial involvement. Furthermore, Mehta et al.^29^ observed that the presence of RV myocardial involvement itself, rather than the extensive LV infarction, is related to increased risk of death, shock and arrhythmia. In order to clarify this question, it would be necessary to assess the magnitude of the LV infarct by means of ^99m^Tc single-photon emission computed tomography (SPECT) and peak creatine kinase (CK), as well as assessing LV function by means of radionuclide angiography (RNA).^29^

The present study is in accordance with the results from previous studies, thus demonstrating that patients with LV myocardial infarction who have RV myocardial involvement are at substantially increased risk of major complications, including death. In the present study, the diagnosis of RV myocardial involvement was based on EKG criteria alone, even though RV myocardial involvement is often considered to be a clinical syndrome in which hypotension and elevated right-side pressure are required to make the diagnosis. However, ST-segment elevation in the V_4_R lead has been shown in previous studies to be highly sensitive and also specific for RV dysfunction in LV acute infarction.^17,30^

Our study suggests that right-side precor-dial leads should be utilized, for all patients presenting ischemic chest pain at emergency services, as the first complementary examination upon patient admission. Routine adoption of this approach will facilitate early identification of patients with RV myocardial involvement who are at high risk of life-threatening complications and who may warrant more aggressive specific treatment.

## CONCLUSIONS

Patients with acute left ventricular infarction who present associated right ventricular infarction are at increased risk of early morbidity and mortality. The adverse prognosis for patients with RV myocardial involvement may be a reflection of more extensive LV infarction.
